# Retraction: Intra-articular injections with either triamcinolone hexacetonide, stanozolol, hylan G-F 20, or a platelet concentrate improve clinical signs in police working dogs with bilateral hip osteoarthritis

**DOI:** 10.3389/fvets.2022.1130041

**Published:** 2023-01-18

**Authors:** Frontiers Editorial Office

Following publication, concerns were raised about similarities between the article cited above in Frontiers in Veterinary Science and Scientific Reports (Alves JC, Santos A, Jorge P, Lavrador C, Carreira LM. Intraarticular triamcinolone hexacetonide, stanozolol, Hylan G-F 20 and platelet concentrate in a naturally occurring canine osteoarthritis model. *Sci Rep*. (2021) 11:3118. doi: 10.1038/s41598-021-82795-z), and PLOS One (Alves JC, Santos A, Jorge P, Lavrador C, Carreira LM. The intra-articular administration of triamcinolone hexacetonide in the treatment of osteoarthritis. Its effects in a naturally occurring canine osteoarthritis model. *PLoS ONE*. (2021) 16:e0245553. doi: 10.1371/journal.pone.0245553).

The three studies share data, results and conclusions to an extent that is inconsistent with Frontiers requirements for the publication of original research.

Following the assessment and agreement of the Field Chief Editor of Frontiers in Veterinary Science, the Specialty Chief Editor of Veterinary Regenerative Medicine and the Specialty Chief Editor of Comparative and Clinical Medicine, and in accordance with Committee on Publication Ethics guidelines, this article is retracted.

The authors do not consent to this retraction.

